# Which factors explain variation in intention to disclose a diagnosis of dementia? A theory-based survey of mental health professionals

**DOI:** 10.1186/1748-5908-2-31

**Published:** 2007-09-25

**Authors:** Robbie Foy, Claire Bamford, Jillian J Francis, Marie Johnston, Jan Lecouturier, Martin Eccles, Nick Steen, Jeremy Grimshaw

**Affiliations:** 1 Institute of Health and Society, Newcastle University, 21 Claremont Place, Newcastle upon Tyne NE2 4AA, UK; 2 Health Services Research Unit, Health Sciences Building, University of Aberdeen, Foresterhill, Aberdeen AB25 2ZD, UK; 3 School of Psychology, College of Life Sciences and Medicine, William Guild Building, University of Aberdeen, Aberdeen AB24 2UB, UK; 4 Ottawa Health Research Institute, 725 Parkdale Avenue, Ottawa ON K1Y 4E9, Canada

## Abstract

**Background:**

For people with dementia, patient-centred care should involve timely explanation of the diagnosis and its implications. However, this is not routine. Theoretical models of behaviour change offer a generalisable framework for understanding professional practice and identifying modifiable factors to target with an intervention. Theoretical models and empirical work indicate that behavioural intention represents a modifiable predictor of actual professional behaviour. We identified factors that predict the intentions of members of older people's mental health teams (MHTs) to perform key behaviours involved in the disclosure of dementia.

**Design:**

Postal questionnaire survey.

**Participants:**

Professionals from MHTs in the English National Health Service.

**Methods:**

We selected three behaviours: Determining what patients already know or suspect about their diagnosis; using explicit terminology when talking to patients; and exploring what the diagnosis means to patients. The questionnaire was based upon the Theory of Planned Behaviour (TPB), Social Cognitive Theory (SCT), and exploratory team variables.

**Main outcomes:**

Behavioural intentions.

**Results:**

Out of 1,269 professionals working in 85 MHTs, 399 (31.4%) returned completed questionnaires. Overall, the TPB best explained behavioural intention. For determining what patients already know, the TPB variables of subjective norm, perceived behavioural control and attitude explained 29.4% of the variance in intention. For the use of explicit terminology, the same variables explained 53.7% of intention. For exploring what the diagnosis means to patients, subjective norm and perceived behavioural control explained 48.6% of intention.

**Conclusion:**

These psychological models can explain up to half of the variation in intention to perform key disclosure behaviours. This provides an empirically-supported, theoretical basis for the design of interventions to improve disclosure practice by targeting relevant predictive factors.

**Trial Registration:**

ISRCTN15871014.

## Background

The early care of people with dementia ideally involves a sensitive and accurate explanation of the diagnosis to individuals and informal carer-givers, and information about the likely prognosis and possible packages of care [1]. Timely disclosure can facilitate decisions about treatment – increasingly important with the advent of therapies to slow disease progression – and allows opportunities to plan family, fiscal and long-term care arrangements. In the context of recognised aspects of quality of care, disclosure therefore needs to be patient-centred and timely [2].

From an ethical perspective, people with dementia have a right to know their diagnosis. Furthermore, many want to know their diagnosis or more information about their illness [3-7]. The majority of people with dementia found it helpful to have been told their diagnosis [3]. Specific benefits include validating their perception that something is wrong [8] and helping them make sense of their experience [5]. Recent qualitative studies have highlighted the range of coping strategies used by people with dementia to adjust to their diagnosis [9-13]. In contrast, lack of information can cause distress and forestall opportunities to engage in grief work to cope with loss.

Yet disclosure practice by healthcare professionals varies widely [14]. The diagnosis is often disclosed to caregivers but not to people with dementia themselves [15]. There is therefore substantial scope for improving professional practice. A considerable body of literature suggests that a range of interventions (e.g., reminder systems, interactive education) can be effective in changing professional behaviour [16]. But there is little empirical evidence on which strategy is most appropriate in the light of a given context or targeted clinical behaviour [17] due to problems understanding the generalisability of the strategies used. One way forward is to use a generalisable framework such as that offered by theory [18-20]. Many factors may influence disclosure: patient characteristics (e.g., age, ability to retain the diagnosis); nature of the dementia (e.g., severity, diagnostic uncertainty); structural factors (e.g., time); and clinician factors (e.g., perceived value of disclosure) [14,21-27]. Some of these may be amenable to change and hence targeted in efforts to improve disclosure practice. Theoretical models of behaviour change allow identification of potentially modifiable factors to target with an intervention [28]. While it would be useful to identify factors that predict professionals' actual disclosure behaviour, there are several problems in measuring behaviour, such as poor recall of events by people with dementia [29]. However, one potentially modifiable factor that can predict actual behaviour is behavioural intention (or motivation).

Behavioural intention is a valid proxy for behaviour predicting 27–28% of the variance in actual behaviour across a wide range of contexts [30,31]. A recent systematic review of the relationship between clinical behaviours and behavioural intention found that the proportion of variance in behaviour explained by intention was of a similar magnitude to that found in the literature relating to non-health professionals [32]. Further, behaviour change rarely occurs in those lacking the intention to change their behaviour [33]. In other words, intention is a necessary but not sufficient condition for action. Knowing whether intentions are low is an important part of identifying barriers to action. It is in this spirit that we used intention as an important proximal determinant of behaviour. Therefore, explaining variation in behavioural intention represents a useful step in efforts to improve disclosure practice, consistent with the initial phases recommended for the development and evaluation of complex interventions [34]. This paper describes the first stages of a larger study to develop an intervention to promote appropriate disclosure [35]. We surveyed members of mental health teams (MHTs) for older people to identify factors that predict their intention to disclose a diagnosis of dementia to patients.

## Methods

### Participants

Eligible participants were members of MHTs for older people from 35 National Health Service (NHS) Trusts in the North of England that provided mental health services and a random sample of Trusts from elsewhere in England. Although disclosure of dementia might predominantly be regarded as the responsibility of specialist old age psychiatrists, other professionals (e.g., community psychiatric nurses, clinical psychologists) have various roles in this process. Therefore, we invited all professionals in each team to participate.

### Selection of theories

We selected two theories, the Theory of Planned Behaviour (TPB) [36] and Social Cognitive Theory (SCT) [37]; both have been rigorously evaluated in other settings and they explain behaviour in terms of factors amenable to change (e.g., beliefs, perceived external constraints). There were economies of measurement inherent in using both theories because of overlapping constructs. According to the TPB, the strength of a behavioural intention is predicted by attitudes towards the behaviour (in this case disclosure), subjective norms based on the perceived views of other individuals or groups (i.e., perceived social pressure), and perceived behavioural control, encompassing beliefs about self-efficacy (an individual's confidence about being able to perform an action) and wider environmental factors that enable or inhibit performance [36]. SCT considers self-efficacy, outcome expectancy (an individual's estimate that a given behaviour will lead to certain outcomes) and individuals' goals in explaining behaviour, including proximal goals (such as intentions) [37].

The above theories are concerned with individual behaviour. Factors such as the lack of clarity of roles and responsibilities within teams may also influence disclosure practice [38]. Therefore we planned to include some exploratory questions around these factors.

### Selection of behaviours

Appropriate disclosure encompasses multiple actions taken by professionals, usually over a period of time, tailored to individuals' receptiveness and information needs. We identified key behavioural components from a literature review, interviews with people with dementia and caregivers, and a consensus panel including a range of professionals and a patient advocate.

We judged that, based on likely length, a theory-based questionnaire could explore up to three specific behaviours. We used a Delphi process to select three behaviours based on the following criteria: covering different stages of the disclosure process; the earlier consensus panel rankings; importance to people with dementia and caregivers; evidence of benefit; and potential for change. The behaviours were:

1. Determining what the patient already knows or suspects about their diagnosis;

2. Using the actual words 'dementia' or 'Alzheimer's disease' when talking to the patient; and

3. Exploring what the diagnosis means to the patient.

### Questionnaire development

Items measuring variables from the TPB and SCT were initially derived from previously recommended scales and items [36,37,39,40] as well as a qualitative analysis of interviews with people with dementia and caregivers. The items and format were then iteratively developed during cognitive interviews with a convenience sample of six mental health professionals.

The main questionnaire constructs are summarised below. The items (Additional File 1) and the full questionnaire in (Additional File 2) are also available. To reduce response set bias, some items were reverse-worded and responses reverse-scored.

1. Behavioural intentions for both the TPB and (as a measure of proximal goals) SCT were measured by two items for each behaviour in the context of a given scenario in which the professional was confident of the diagnosis of dementia.

2. Attitude items related to expected consequences (for both patient and professional) of performing the behaviour. Three items measured the emotional impact on professionals of performing each disclosure behaviour (hereafter referred to as 'emotional attitude'). The seven to ten attitude items for each behaviour also served to measure outcome expectancies for SCT.

3. Subjective norm comprises normative beliefs (about whether specific reference groups or individuals think a person should perform a behaviour) weighted by the person's motivation to comply with these views. Three items assessed normative beliefs for each behaviour as professionals may perceive different levels of approval or disapproval from a range of groups (e.g., other team colleagues, patients). The three items measuring motivation to comply with these sources of pressure related to disclosure of a diagnosis of dementia in general. Answers to these items provided weights (i.e., multipliers) for normative belief scores. Weighted normative beliefs were summed to produce subjective norm scores and standardised to a one to seven score to facilitate comparisons with other scores. A fourth subjective norm item included the idea of motivation to comply in the form of specifying 'people who are important to me professionally' and so weighting was not required [36].

4. Perceived behavioural control. There were three control items per behaviour, using recommended stems [36]. e.g., 'It is easy to ...'; 'I feel I have the skills to ....'; 'The decision to ... is beyond my control'.

5. Self-efficacy. There were four to eight self-efficacy items per behaviour, specifying situations where professionals might feel different levels of confidence in their ability to enact each behaviour.

6. Team role. We included the following exploratory items as they may influence intention to perform disclosure behaviours:

a) Perceived reliability/role of colleagues, e.g., "I can rely on my colleagues in my mental health team to use the actual words 'dementia' or 'Alzheimer's Disease' when talking to the patient".

b) Role responsibility. Items concerning which team members were responsible for each behaviour: psychiatrist; social worker; clinical psychologist; community psychiatric nurse; occupational therapist; care or nursing assistant; in-patient or day hospital nurse; or other (giving details). These provided data about whether each respondent's own professional group was regarded as responsible for the behaviour and, when aggregated for each team, the number of professional groups in each team perceived as being responsible for the behaviour.

### Survey administration

We ascertained the composition of MHTs from local contacts, usually service managers. We then wrote to all professionals via these contacts and asked those who agreed to participate to complete an 'opt-in' form. All potential participants were offered a small financial incentive (a £20 gift voucher), enclosed with the questionnaire subsequently sent out. We asked respondents to complete questionnaires independently (i.e., not together in teams). We posted up to three reminders to non-respondents who had opted in earlier.

### Sample Size

Power calculations for multiple regression analysis depend on the number of cases per predictor variable. A minimum sample size of 50 + 8 m, where m is the number of predictor variables, is recommended for testing the multiple correlation, and 104 + m for testing individual predictors [41,42]. With approximately 10 predictor variables for each behaviour, minimum sample sizes of 130 and 114 subjects were required to test the multiple correlation and individual predictors respectively. Taking the larger figure as the target sample size and conservatively assuming a 30% response rate from individuals, we planned to approach an estimated 420 individuals from 120 MHTs.

### Analysis

The internal reliability of the constructs was assessed using Cronbach's alpha coefficient and by considering the correlation of each item with the construct score calculated without the inclusion of that item (item-total correlation). A figure of 0.6 was specified as an appropriate threshold below which internal reliability was considered to be unsatisfactory. In these cases, either a subset of items was identified that did have adequate reliability or a single item was selected on the basis of face validity. We compared differences in mean construct scores between the three behaviours using the variance ratio test. Pearson product moment correlation coefficients were used to examine the bivariate relationships between constructs.

The relationships between intention and TPB, SCT, and team constructs were investigated using multiple regression with intention specified as the dependent variable. This was done in two stages. In the first stage, the relationship between intention and the set of constructs from each theoretical model was assessed separately. For each variable set the predictor variables were added using a stepwise procedure. The variable most highly correlated with intention was added first. On subsequent steps the variable explaining the greatest amount of the residual variation was added provided that the improvement in the fit of the model was significant at the 5% level.

In the second stage, all constructs that significantly predicted intention in parallel regression analyses for the three behaviours were simultaneously entered into a stepwise regression analysis.

### Ethical approval

The study was approved by the Multi-Centre Research Ethics Committee for Scotland and by the Research and Development offices of the participating NHS Trusts.

## Results

### Response rates

Out of the 35 trusts approached, four did not provide team information or distribute opt-in letters and eight were excluded due to delays in obtaining research governance approval. In the remaining 23 trusts, we identified 114 MHTs for older people and 1,269 individual professionals. Out of these individuals, 420 (33.1%) from 85 teams opted in and 399 (31.4%) returned completed questionnaires (Table 1). The number of teams per trust professionals per team was higher than anticipated, contributing to a larger number of responses than we had anticipated.

**Table 1:** Response rates by professional group

	Identified	Opted in^a^	Completed questionnaire^a^
MH teams	114	85 (74.6%)	85 (74.6%)
Doctors	185	54 (29.2%)	51 (27.6%)
Nurses	535	206 (38.5%)	198 (37.0%)
Professions allied to medicine	246	95 (38.6%)	89 (36.2%)
Social workers	116	28 (24.1%)	27 (23.3%)
Support workers (health and social care)	130	37 (28.5%)	34 (26.2%)
All mental health team members	1269^b^	420 (33.1%)	399 (31.4%)

^a ^For MHTs, data refer to the number of teams where at least one professional opted in or completed a questionnaire

^b ^This is not equal to the sum of different types of professionals since information on team composition was not available for all MHTs.

### Psychometric properties of measured constructs

For the TPB variables, measures relating to intention, emotional attitudes and subjective norms achieved acceptable internal consistency (alpha ≥ 0.6) for all three behaviours (Table 2). For other constructs, removal of some items improved internal consistency. (Removed items are indicated in Additional File 1.)

**Table 2:** Descriptive and psychometric statistics for each of the psychological and team variables for the three disclosure behaviours.

**Variable**	**Behaviour**									**Overall variance ratio test of a difference between behaviours**			
	**Exploring what patient already knows or suspects **(n = 398)			**Use of explicit terminology **(n = 387)			**Exploring what the diagnosis means to the patient **(n = 385)						

	No. items	Mean^c ^(SD)	Alpha	No. items	Mean^c ^(SD)	Alpha	No. items	Mean^c ^(SD)	Alpha	**F**	**D1**	**D2**	**P**
TPB constructs													
Intention^a^	2	5.72 (1.17)	0.85	2	4.66 (1.47)	0.91	2	5.41 (1.32)	0.92	105.8	2	769	0.000
Emotional attitude	3	5.44 (1.30)	0.79	3	5.18 (1.27)	0.76	3	5.17 (1.31)	0.79	10.0	2	772	0.000
Attitude	3	6.24 (0.86)	0.73	6	4.84 (0.93)	0.77	6	5.53 (0.88)	0.73	424.8	2	763	0.000
Subjective norm	4	5.38 (0.99)	0.80	4	4.52 (1.12)	0.83	4	5.27 (1.09)	0.87	101.7	2	744	0.000
PBC	1	5.42 (1.75)	n/a	1	5.73 (1.42)	n/a	3	5.28 (1.08)	0.67	6.9	2	772	0.001
SCT constructs													
Self efficacy	4	5.02 (0.89)	0.61	8	4.26 (1.02)	0.84	5	5.12 (0.95)	0.79	180.7	2	767	0.000
Outcome expectancies	6	5.85 (0.84)	0.70	9	4.95 (0.85)	0.78	9	5.41 (0.84)	0.76	231.2	2	751	0.000
Team variables													
Perceived reliability of colleagues	1	5.02 (1.50)	n/a	1	4.50 (1.42)	n/a	1	5.15 (1.42)	n/a	39.9	2	772	0.000
Perceived role^b^	1	74.9^d ^(43.4)	n/a	1	68.2^d ^(46.6)	n/a	1	76.9^d ^(42.2)	n/a	14.3	2	796	.000
Number of professional groups^e^	1	4.04 *** (1.92)	n/a	1	3.79 *** (1.98)	n/a	1	3.89*** (1.87)	n/a	3.8	2	796	.024

(^a ^Intention was also used to measure proximal goals for SCT; ^b ^Whether respondent believed own professional group was responsible for behaviour. Responses were coded as a binary item with scores of either 0 or 1; ^c ^Based on a possible range of 1–7 with higher scores indicating a stronger intention to perform the behaviour, etc; ^d ^Percentage of respondents agreeing that their roles included performing this behaviour; ^e^Number of professional groups in each team perceived as being responsible for the behaviour; *Significant p < 0.05; **significant p < 0.01; ***significant p < 0.001).

As we had no *a priori *basis for combining the exploratory team items as a single construct, we did not attempt reliability analyses for them.

### Descriptive data

Table 2 shows the mean values for each of the five psychological variables for the three disclosure behaviours. Mean behavioural intention significantly differed between the three behaviours (F = 105.80; df = 2; p < 0.001), being highest for determining what the patient already knows and lowest for the use of explicit terminology.

The other mean construct scores for the TPB, SCT and team variables also varied significantly between the three behaviours. The following mean scores were all lowest for the use of explicit terminology: attitude; subjective norm; self-efficacy and outcome expectancies. In contrast, mean PBC was highest for the use of explicit terminology.

For the team variables, approximately four professional groups were involved in each behaviour. Fewer (68.2%) respondents considered that using explicit terminology was consistent with their roles compared with determining what the patient knows and exploring the meaning of the diagnosis (74.9% and 76.9% respectively; F = 14.28; p < 0.001). Respondents reported being less able to rely on other colleagues to use explicit terminology compared with the other two behaviours (F = 39.86; p < 0.001).

#### Correlations

For all three behaviours, all psychological variables were significantly correlated (Table 3). The high correlations between the TPB attitude and SCT outcome expectancy are due to overlapping items.

**Table 3:** Correlations between psychological variables (n = 398; TPB constructs are behavioural intention, emotional attitude, attitude, subjective norm and perceived behavioural control. SCT constructs are behavioural intention (as a proxy for proximal goals), self-efficacy and outcome expectancies.)

	Behavioural intention	Emotional attitude	Attitude	Subjective norm	Perceived behavioural control	Self efficacy
**Explore what patient already knows or suspects**						
Behavioural intention	-					
Emotional attitude	0.183***	-				
Attitude	0.296***	0.187***	-			
Subjective norm	0.485***	0089*	0.328***	-		
Perceived behavioural control	0.269***	0.248***	0.235***	0.126*	-	
Self efficacy	0.465***	0.257***	0.211***	0.388***	0.185**	-
Outcome expectancies	0.293***	0.863***	0.657***	0.236***	0.294***	0.318***
**Use of explicit terminology**						
Behavioural intention	-					
Emotional attitude	0.368***	-				
Attitude	0.634***	0.335***	-			
Subjective norm	0.627***	0.217***	0.494***	-		
Perceived behavioural control	0.157**	0.231***	0.164**	0.117*	-	
Self efficacy	0.550***	0.342***	0.469***	0.472***	0.147**	-
Outcome expectancies	0.635***	0.734***	0.885***	0.465***	0.234***	0.498***
**Explore what diagnosis means to patient**						
Behavioural intention	-					
Emotional attitude	0.294***	-				
Attitude	0.367***	0.321***	-			
Subjective norm	0.603***	0.284***	0.358***	-		
Perceived behavioural control	0.583***	0.510***	0.356***	0.441***	-	
Self efficacy	0.561***	0.332***	0.494***	0.523***	0.553***	-
Outcome expectancies	0.411***	0.747***	0.869***	0.404***	0.515***	0.520***

*Significant p < 0.05; **significant p < 0.01; ***significant p < 0.001

In general, respondents reported greater ability to rely on colleagues to perform a disclosure behaviour where they thought that a greater number of professional groups were responsible for the behaviour (correlation coefficients 0.122, p = 0.02 for determining what patient knows; 0.259, p < 0.001 for use of explicit terminology; 0.150, p = 0.003 for exploring meaning of the diagnosis).

### Prediction of intention

For exploring what the patient already knows, the TPB variables of subjective norm, perceived behavioural control, emotional attitude and attitude explained 29.4% of behavioural intention (Table 4). In comparison, SCT explained 24.2% of intention whilst the team variables explained 15.5%. When all constructs were combined, taking into account the overlap between the TPB and SCT constructs, the prediction of intention modestly improved to 35.6%.

**Table 4:** Regression analyses for the three disclosure behaviours.

**Behaviour**	**Model**	**Predictor variables**	**Standardised regression coefficient**	**R^2 ^(%)**
**Explore what patient already knows or suspects (n = 373)**	**TPB constructs**	Subjective norm	0.424	23.5***
		Perceived behavioural control	0.161	4.0***
		Emotional attitude	0.094	1.1*
		Attitude	0.098	0.8*
		*Total R*^2^		*29.4*
	**SCT constructs**	Self efficacy	0.417	22.0***
		Outcome expectancies	0.158	2.2**
		*Total R*^2^		*24.2*
	**Team variables**	Rely on colleagues	0.384	13.1***
		Perceived role	0.140	1.2**
		Number of professional groups	-0.113	1.2*
		*Total R*^2^		*15.5*
	**Combined constructs**	Subjective norm	0.334	23.5***
		Perceived behavioural control	0.213	4.0***
		Perceived reliability of colleagues	0.252	4.6***
		Outcome expectancies	0.145	1.9**
		Number of professional groups responsible for behaviour	-0.128	1.6**
		*Total R*^2^		*35.6*

**Use explicit terminology (n = 366)**	**TPB constructs**	Subjective norm	0.407	38.8***
		Attitude	0.374	13.1***
		Emotional attitude	0.143	1.8***
		*Total R*^2^		*53.7*
	**SCT constructs**	Outcome expectancies	0.470	40.1***
		Self efficacy	0.316	7.4***
		*Total R*^2^		*47.5*
	**Team variables**	Rely on colleagues	0.566	37.6***
		Perceived role	0.220	4.6***
		*Total R*^2^		*42.2*
	**Combined constructs**	Outcome expectancies	0.422	40.1***
		Perceived reliability of colleagues	0.284	15.8***
		Subjective norm	0.183	3.7***
		Self efficacy	0.154	1.8***
		Perceived role	0.127	1.3***
		Emotional attitude	-0.133	0.8**
		*Total R*^2^		*63.5*

**Explore what diagnosis means to patient (n = 371)**	**TPB constructs**	Subjective norm	0.434	36.4***
		Perceived behavioural control	0.389	12.2***
		*Total R2*		*48.6*
	**SCT constructs**	Self efficacy	0.470	29.4***
		Outcome expectancies	0.149	1.7**
		*Total R2*		*31.1*
	**Team variables**	Rely on colleagues	0.349	10.8***
		Perceived role	0.269	7.2***
		*Total R2*		*18.0*
	**Combined constructs**	Subjective norm	0.334	36.3***
		Perceived behavioural control	0.296	12.3***
		Self efficacy	0.161	1.9***
		Perceived role	0.127	1.2**
		Perceived reliability of colleagues	0.109	1.0**
		*Total R2*		*52.7*

p < 0.05; ** p < 0.01; *** p < 0.001. These significance levels are associated with the increase in R^2 ^as explanatory variables are added incrementally to the regression models.

For the use of explicit terminology, the TPB variables of subjective norm, perceived behavioural control, emotional attitude and attitude explained 53.7% of intention. Notably, both SCT and two of the team variables (perceived role and being able to rely on colleagues) also explained high proportions of intention (47.5% and 42.2% respectively). Combining all constructs improved prediction to 63.5%.

For exploring what the diagnosis means to the patient, the TPB variables of subjective norm and perceived behavioural control explained 48.6% of intention. The SCT explained 31.1% of intention, whilst the team variables explained 18%. The combined constructs model added little to prediction (52.7%).

## Discussion

Appropriate disclosure of dementia requires a number of interrelated steps and multi-disciplinary input. Most mental health professionals surveyed recognised their roles in and had positive intentions towards performing three disclosure behaviours. The TPB explained between 28.6% and 53.3% of variation in intentions, with subjective norm consistently representing an important explanatory variable. Intention to use explicit terminology was also explained by attitude but – unlike both other behaviours – not by perceived behavioural control.

The TPB tended to explain a greater proportion of variance in intentions than constructs from SCT and the team variables. This was not simply due to the greater number of predictors (four compared with two for SCT and three for team variables): taking only the first two predictor variables from each model, the TPB still accounted for more variance in each behaviour than either of the other two theoretical approaches. The TPB also was relatively parsimonious compared with the regression model which combined all constructs and modestly improved prediction. We did not conduct an analysis that allows the TPB and SCT to compete directly. Instead we added sets of predictors hierarchically, so for the overlapping constructs (e.g., self-efficacy with perceived behavioural control and outcome expectancies with attitude), beta weights were expected to reflect both some of the predictive value of each variable and also the order of entry of variables.

The team variables accounted for considerably more variance in the use of explicit terminology compared with both other behaviours, with the lone variable of being able to rely on colleagues explaining over a third of intention (37.4%). However, this variable explained less variation in the combined constructs model, suggesting that its effects may be mediated through other predictors. For example, subjective norm for the use of explicit terminology may incorporate aspects of teamwork (i.e., 'Members of my MHT would approve of my using the actual words 'dementia' or 'Alzheimer's disease' when talking to the patient.')

Our findings have several implications for improving the quality of disclosure practice. On a 7-point scale, mean intention was 5.72 (SD 1.17) for determining what the patient already knows. This suggests modest scope for further improving intention (and hence actual behaviour) [43]. According to results based on the TPB, interventions predominantly targeting subjective norm and perceived behavioural control may have the greatest impact in changing intention (as these are the strongest predictors of intention), with lesser effects expected by targeting attitudes. While there is no guarantee that changing a significant predictor will result in changed behaviour, this approach uses an evidence base in a systematic way to select intervention components and thus to move forward from correlational designs and test plausible hypotheses using experimental designs.

For the use of explicit terminology, the mean intention score was 4.66 (SD 1.47) and lowest out of the three behaviours. This suggests relatively greater potential exists for changing intention and hence practice. Interventions targeting subjective norm, attitudes relating to outcomes for the patient and professional and, possibly, emotional attitude may represent key targets for intervention. However, the team variables – whether professionals perceived they could rely on colleagues to use explicit terminology and whether professionals also believed this behaviour to be compatible with their own roles – explained over 40% of variation in intention. Interventions around clarifying and revising team roles may offer promising means of changing intention, potentially also operating via influencing subjective norms.

For exploring what the diagnosis means to the patient, the mean intention score of 5.41 (SD 1.32) suggests modest scope for changing intention and hence behaviour. Interventions targeting subjective norms and perceived behavioural control offer the most promising means of achieving this.

Consistent with the predictions of SCT, both self-efficacy and outcome expectancies explained intention for all three behaviours, albeit less than the TPB variables did. Given the overlap with attitude, the pattern of outcome expectancies predicting intention across the three behaviours is expected. However, strategies to improve disclosure practice should also consider means of enhancing self-efficacy.

Earlier studies have investigated reasons why mental health professionals do or do not disclose dementia [3-6]. Their methods have several limitations which have been addressed by this study. First, the interpretation of these and studies in other contexts that attempt to understand clinical practice or improve quality of care is often hampered by the lack of an underlying robust theoretical model that explains behaviour in terms of cognitive factors. The assumption that clinical practice is a form of human behaviour and can be described in terms of general theories relating to human behaviour offers the basis for a generalisable framework for understanding and developing interventions to change professional behaviour. Second, professionals' reported reasons for their actions may not explain their actual practice. Social desirability bias may explain why respondents to our survey rated their own intentions, attitudes and beliefs in a relatively favourable light. However, other studies have demonstrated that measuring behavioural intention using similar methods can usefully predict actual behaviour [30-32]. Third, many previously reported barriers to appropriate disclosure are less amenable to change within the present healthcare context and resources, e.g., severity of dementia or lack of time. We examined potentially modifiable variables, namely, behavioural intention and its predictors.

One limitation to this study was the relatively low response rate (31%) from the eligible sample. This was probably partly attributable to the staged 'opt-in' process necessary to comply with data protection legislation. Most (95%) professionals who opted in to do the survey did complete questionnaires. However, external validity may be undermined if respondents differed systematically from non-respondents, e.g., had more positive attitudes towards disclosure.

We did not explore the contribution of professional roles in predicting intention, largely because it was beyond the scope of this paper. We are presently addressing this issue in another survey, within a subsequent stage of this programme of work, using a much larger sample which will allow better precision for exploring differences between professional groups.

We do not claim that other intervention strategies, such as restructuring teams or care delivery processes, would be of lesser effectiveness than any intervention developed using the approach we describe. Such environmental or organisational changes may also change underlying psychological factors – so that changes in beliefs or attitudes following 'forced' changes in practice. The perspective of social cognitive theories is that external influences on behaviour are mediated through perceptions of individuals (i.e., the predictor variables in the theories we have used). They do not claim that external influences do not 'drive' behaviour; merely that they are unlikely to drive behaviour without the awareness of the person who is behaving. We have made the assumption that the disclosure behaviours under investigation are enacted voluntarily, with low levels of automatism.

Our findings provide an empirically-supported, theoretical basis for the design of interventions to improve the quality of disclosure practice by targeting relevant predictive factors. It is uncertain whether the causal relationships predicted by theory would be seen in a subsequent intervention study. For example, the factors that determine current behaviour may differ from those that determine change in behaviour. The next step is therefore to evaluate whether interventions targeting the key predictive variables identified in this study do increase intention.

## Competing interests

Martin Eccles is Co-Editor in Chief of Implementation Science and Robbie Foy is Associate Editor; all editorial decisions on this article were made by Co-Editor in Chief Brian Mittman.

## Authors' contributions

ME and MJ conceived the original idea for this study. ME, RF, CB, MJ, NS and JG obtained grant funding. JL, CB and RF led the conduct of the survey. NS and CB analysed the survey data which was interpreted by all authors. RF and CB wrote the first draft of the manuscript and all authors participated in subsequent revisions. All authors read and approved the final manuscript.

## Supplementary Material

Additional file 1 Questionnaire items. Questionnaire constructs and items by disclosure behaviour. Click here for file

Additional file 2 Talking to people with dementia about their diagnosis – can we do it better? The survey questionnaire. Click here for file
