# Supplementary material for: Which factors explain variation in intention to disclose a diagnosis of dementia? A theory-based survey of mental health professionals
Source: Implement Sci. 2007 Sep 25;2:31. doi: 10.1186/1748-5908-2-31 (PMC2042985; doi:10.1186/1748-5908-2-31)
Supplement: Additional file 1 — Questionnaire items. Questionnaire constructs and items by disclosure behaviour. [file 1748-5908-2-31-S1.doc]

**Appendix 1. Questionnaire constructs and items by disclosure behaviour.** Note: Items in italic were omitted from the calculated construct scores prior to the final regression analysis.

A. Finding out what the patient already knows or suspects about their diagnosis.

| Construct | Items | Response format (on 1-7 Likert scale; R = reverse scored) |
| --- | --- | --- |
| Behavioural intention | I intend to find out what the patient already knows or suspects | Strongly disagree – strongly agree |
|  | In my practice I expect to find out what the patient already knows or suspects |  |
| Attitude | I think that, overall, finding out what the patient already knows or suspects is… | Harmful to the patient – beneficial to the patient |
|  |  | The wrong thing to do – the right thing to do |
|  |  | *Not distressing to the patient – distressing to the patient (R)* |
|  |  | A poor use of my time – a good use of my time |
| Emotional attitude | I would feel uncomfortable whilst finding out what the patient already knows or suspects | Strongly disagree – strongly agree (all R) |
|  | I would feel out of my depth whilst finding out what the patient already knows or suspects |  |
|  | I would find it upsetting to find out what the patient already knows or suspects |  |
| Subjective norm (the first three are normative beliefs) | Generally, patients with dementia think that I should find out what they already know or suspect | Strongly disagree – strongly agree |
|  | Generally, carers and relatives of patients with dementia think that I should find out what the patient already knows or suspects |  |
|  | Members of my Mental Health Team would approve of my finding out what the patient already knows or suspects |  |
|  | Other people who are important to me professionally would approve of my finding out what the patient already knows or suspects |  |
| Subjective norm (motivation to comply with various sources of social pressure)* | Thinking about my role in the process of disclosing a diagnosis of dementia … what patients with dementia think I should do is very important to me |  |
|  | … what carers and relatives of patients with dementia think I should do is very important to me |  |
|  | … what members of my Mental Health Team think I should do is very important to me |  |
| Perceived behavioural control | The decision whether or not to find out what the patient already knows or suspects is beyond my control | Strongly disagree – strongly agree (R) |
|  | *I feel that I have the skills I need to find out what the patient already knows or suspects* | Strongly disagree – strongly agree |
|  | *It is easy for me to find out what the patient already knows or suspects* |  |
| Self-efficacy | I am confident that I can find out what the patient already knows or suspects ….when I have sufficient time with the patient |  |
|  | ….when a carer or relative is present |  |
|  | ….when the patient is mildly impaired |  |
|  | ….when the patient is severely impaired |  |

*These same three items were also used to measure motivation to comply for the other two behaviours.

B. Using the actual words ‘dementia’ or ‘Alzheimer’s disease’ when talking to the patient

| Construct | Items | Response format (on 1-7 Likert scale; R = reverse scored) |
| --- | --- | --- |
| Behavioural intention | I intend to use the actual words ‘dementia’ or ‘Alzheimer’s disease’ when talking to the patient | Strongly disagree – strongly agree |
|  | In my practice I expect to use the actual words ‘dementia’ or ‘Alzheimer’s disease’ when talking to the patient |  |
| Attitude | I think that, overall, using the actual words ‘dementia’ or ‘Alzheimer’s disease’ when talking to the patient … | Is harmful to the patient – beneficial to the patient |
|  |  | Is the wrong thing to do – the right thing to do |
|  |  | *Is not distressing to the patient – distressing to the patient I* |
|  |  | Is a poor use of my time – a good use of my time |
|  |  | Results in patient being stigmatised – does not result in patient being stigmatised |
|  |  | Makes it easier for the patient to understand what is happening to them – makes it more difficult for patient to understand what is happening to them (R) |
|  |  | Discourages the patient from planning for the future – encourages the patient to plan for the future |
| Emotional attitude | I would feel uncomfortable whilst using the words ‘dementia’ or ‘Alzheimer’s disease’ when talking to the patient | Strongly disagree – strongly agree (all R) |
|  | I would feel out of my depth whilst using the words ‘dementia’ or ‘Alzheimer’s disease’ when talking to the patient |  |
|  | I would find it upsetting to use the words ‘dementia’ or ‘Alzheimer’s disease’ when talking to the patient |  |
| Subjective norm (the first three are normative beliefs) | Generally, patients with dementia think that I should use the actual words ‘dementia’ or ‘Alzheimer’s disease’ when talking to them | Strongly disagree – strongly agree |
|  | Generally, carers and relatives of patients with dementia think that I should use the actual words ‘dementia’ or ‘Alzheimer’s disease’ when talking to the patient |  |
|  | Members of my Mental Health Team would approve of my using the actual words ‘dementia’ or ‘Alzheimer’s disease’ when talking to the patient |  |
|  | Other people who are important to me professionally would approve of my using the actual words ‘dementia’ or ‘Alzheimer’s disease’ when talking to the patient |  |
| Perceived behavioural control | The decision whether or not to use the actual words ‘dementia’ or ‘Alzheimer’s disease’ when talking to the patient is beyond my control | Strongly disagree – strongly agree (R) |
|  | *It is easy for me to use the actual words ‘dementia’ or ‘Alzheimer’s disease’ when talking to the patient* | Strongly disagree – strongly agree |
| Self-efficacy | I am confident that I can use the words ‘dementia’ or ‘Alzheimer’s disease’ when talking to the patient …when the patient thinks that their problems are just due to old age |  |
|  | …when I have sufficient time with the patient |  |
|  | …when a carer or relative is present |  |
|  | …when the patient does not want to know the diagnosis |  |
|  | …when the patient is mildly impaired |  |
|  | ...when the patient is severely impaired |  |
|  | …when the carer has requested that the diagnosis be withheld |  |
|  | …when appropriate support for the patient is available shortly afterwards |  |

C. Exploring what the diagnosis means to the patient

| Construct | Items | Response format (on 1-7 Likert scale; R = reverse scored) |
| --- | --- | --- |
| Behavioural intention | I intend to explore what the diagnosis means to the patient | Strongly disagree – strongly agree |
|  | In my practice I expect to explore what the diagnosis means to the patient |  |
| Attitude | I think that, overall, exploring what the diagnosis means to the patient is … | Is harmful to the patient – beneficial to the patient |
|  |  | Is the wrong thing to do – the right thing to do |
|  |  | *Is not distressing to the patient – distressing to the patient (R)* |
|  |  | Is a poor use of my time – a good use of my time |
|  |  | Results in patient being stigmatised – does not result in patient being stigmatised |
|  |  | Makes it easier for the patient to understand what is happening to them – makes it more difficult for patient to understand what is happening to them (R) |
|  |  | Discourages the patient from planning for the future – encourages the patient to plan for the future |
| Emotional attitude | I would feel uncomfortable whilst exploring what the diagnosis means to the patient | Strongly disagree – strongly agree (all R) |
|  | I would feel out of my depth whilst exploring what the diagnosis means to the patient |  |
|  | I would find it upsetting to explore what the diagnosis meant to the patient |  |
| Subjective norm (the first three are normative beliefs) | Generally, patients with dementia think that I should explore what the diagnosis means to them | Strongly disagree – strongly agree |
|  | Generally, carers and relatives of patients with dementia think that I should explore what the diagnosis means to the patient |  |
|  | Members of my Mental Health Team would approve of my exploring what the diagnosis means to the patient |  |
|  | Other people who are important to me professionally would approve of my exploring what the diagnosis means to the patient |  |
| Perceived behavioural control | The decision whether or not to explore what the diagnosis means to the patient is beyond my control | Strongly disagree – strongly agree (R) |
|  | I feel that I have the skills I need to explore what the diagnosis means to the patient | Strongly disagree – strongly agree |
|  | It is easy for me to explore what the diagnosis means to the patient |  |
| Self-efficacy | I am confident that I can explore what the diagnosis means to the patient … when I have sufficient time with the patient |  |
|  | … when a carer or relative is present |  |
|  | … when the patient is mildly impaired |  |
|  | … when the patient is severely impaired |  |
|  | … when appropriate support for the patient is available shortly afterwards |  |
