# Supplementary material for: Which factors explain variation in intention to disclose a diagnosis of dementia? A theory-based survey of mental health professionals
Source: Implement Sci. 2007 Sep 25;2:31. doi: 10.1186/1748-5908-2-31 (PMC2042985; doi:10.1186/1748-5908-2-31)
Supplement: Additional file 2 — Talking to people with dementia about their diagnosis – can we do it better? The survey questionnaire. [file 1748-5908-2-31-S2.doc]

| Code |  |  |  |  |  |  |
| --- | --- | --- | --- | --- | --- | --- |

**CONFIDENTIAL**

**Talking to people with dementia about their diagnosis – can we do it better?**

**Questionnaire for Older Age Mental Health Teams**

This questionnaire explores factors that influence whether and how members of old age mental health teams tell patients with dementia their diagnosis. This is not a test. For most questions there are no right or wrong answers and all responses will be treated in the strictest confidence.

We recognise that disclosure is a complex process. This questionnaire covers a few selected but important actions within that process. These are:

- Finding out what the patient already knows or suspects
- Using the words ‘dementia’ or ‘Alzheimer’s disease’ when talking to the patient
- Exploring what the diagnosis means to the patient

All of the questions relate to situations where you are confident of the diagnosis of dementia. Obviously the process of disclosure needs to be adapted to each individual patient with dementia so these actions may not be appropriate in every case. We also appreciate that these actions may not form part of your role within the team, for example, because of team policy or because you delegate this to somebody else. The questions also explore this issue.

Some of the questions may seem repetitive or similar, but we do ask that you try to answer every question. The questionnaire should take about 15 minutes to complete. Please circle whichever number best describes your views or experience. Try not to take too long over each response – what comes to mind first is more likely to reflect what you believe.

## Thank you very much for your time

If you wish to find out more about this survey please contact:

| Jan Lecouturier  Research Associate | 0191 222 5629 | [Jan.Lecouturier@ncl.ac.uk](mailto:Jan.Lecouturier@ncl.ac.uk) |
| --- | --- | --- |
| Claire Bamford  Senior Research Associate | 0191 222 7047 | [c.h.bamford@ncl.ac.uk](mailto:c.h.bamford@ncl.ac.uk) |
| Robbie Foy  Senior Lecturer in Primary Care | 0191 222 7214 | [R.C.Foy@ncl.ac.uk](mailto:R.Foy@ncl.ac.uk) |

|  | |
| --- | --- |
| ***SECTION A* FINDING OUT WHAT THE PATIENT ALREADY**  **KNOWS OR SUSPECTS ABOUT THEIR DIAGNOSIS**  **These questions are about the way you prepare the patient for the diagnosis**  **This might include informal conversations or contacts with patients** | |
|  |  |
|  | **I think that, overall, finding out what the patient already knows or suspects is…** |
|  |  |

| 1 | …harmful to the patient | | |  | 1 | 2 | 3 | 4 | 5 | 6 | 7 | beneficial to the patient |
| --- | --- | --- | --- | --- | --- | --- | --- | --- | --- | --- | --- | --- |
|  |  |  |  |  |  |  |  |  |  |  |  |  |
|  |  |  |  |  |  |  |  |  |  |  |  |  |
| 2 | …the wrong thing to do | | |  | 1 | 2 | 3 | 4 | 5 | 6 | 7 | the right thing to do |
|  |  |  |  |  |  |  |  |  |  |  |  |  |
|  |  |  |  |  |  |  |  |  |  |  |  |  |
| 3 | …not distressing to the  patient | | |  | 1 | 2 | 3 | 4 | 5 | 6 | 7 | distressing to the patient |
|  |  |  |  |  |  |  |  |  |  |  |  |  |
|  |  |  |  |  |  |  |  |  |  |  |  |  |
| 4 | …a poor use of my time | | |  | 1 | 2 | 3 | 4 | 5 | 6 | 7 | a good use of my time |
|  |  | |  |  |  |  |  |  |  |  |  |  |
|  |  | |  |  |  |  |  |  |  |  |  |  |
|  |  | |  |  |  |  |  |  |  |  |  |  |

|  | | |  | | | |  | |  | **Strongly Disagree** | | | | | |  | | | | |  | |  | | | | **Strongly**  **Agree** | | | | | |  | | |
| --- | --- | --- | --- | --- | --- | --- | --- | --- | --- | --- | --- | --- | --- | --- | --- | --- | --- | --- | --- | --- | --- | --- | --- | --- | --- | --- | --- | --- | --- | --- | --- | --- | --- | --- | --- |
|  | | |  | | | |  | |  | | | | | | | | | | | | | | | | | |  | | | | | |  | | |
| 5 | | Generally, patients with dementia think that I should find out what they already know or suspect | | | | | | | |  | 1 | | | 2 | | | | 3 | | 4 | | 5 | | | 6 | | | | 7 | |  | | | | |
|  | |  | | | |  | |  | | | | | | | | | | | | | | | | | | | |  | | | | | |  | |
|  | |  | | | |  | |  | | | | | | | | | | | | | | | | | | | |  | | | | | |  | |
| 6 | | Generally, carers and relatives of patients with dementia think that I should find out what the patient already knows or suspects | | | | | | | |  | | 1 | | | 2 | | | | 3 | | 4 | | 5 | | | 6 | | | | 7 | |  | | | |
|  | |  | | | |  | |  | | | | | | | | | | | | | | | | | | | |  | | | | | |  | |
|  | |  | | | |  | |  | | | | | | | | | | | | | | | | | | | |  | | | | | |  | |
| 7 | Members of my Mental Health Team would approve of my finding out what the patient already knows or suspects | | | | | | | | |  | 1 | | 2 | | | | 3 | | | 4 | | 5 | | | 6 | | | | 7 | |  | | | | |
|  | |  | | | |  | |  | | | | | | | | | | | | | | | | | | | |  | | | | | |  | |
|  | |  | | | |  | |  | | | | | | | | | | | | | | | | | | | |  | | | | | |  | |
| 8 | Other people who are important to me professionally would approve of my finding out what the patient already knows or suspects | | | | | | | | |  | 1 | | 2 | | | | 3 | | | 4 | | 5 | | | 6 | | | | 7 | |  | | | | |
|  | | |  |  |  | | | | | | | | | | | | | | | | | | |  | | | | | | | | | | |  |
|  | | |  |  |  | | | | | | | | | | | | | | | | | | |  | | | | | | | | | | |  |
|  | | |  |  |  | | | | | | | | | | | | | | | | | | |  | | | | | | | | | | |  |
|  | | |  |  |  | | | | | | | | | | | | | | | | | | |  | | | | | | | | | | |  |

| **In relation to finding out what the patient already knows or suspects about their diagnosis** | | | | |
| --- | --- | --- | --- | --- |
|  |  |  |  |  |

|  | | |  | | |  | |  | **Strongly Disagree** | | | | | | | | | | | |  | | | | | | | |  | | | |  | | | | | | **Strongly**  **Agree** | | | | | | | | | |  | |
| --- | --- | --- | --- | --- | --- | --- | --- | --- | --- | --- | --- | --- | --- | --- | --- | --- | --- | --- | --- | --- | --- | --- | --- | --- | --- | --- | --- | --- | --- | --- | --- | --- | --- | --- | --- | --- | --- | --- | --- | --- | --- | --- | --- | --- | --- | --- | --- | --- | --- | --- |
|  | | |  | | |  | |  | | | | | | | | | | | | | | | | | | | | | | | | | | | | | | |  | | | | | | | | | |  | |
| 9 | | The decision whether or not to find out what the patient already knows or suspects is beyond my control | | | | | | |  | | | 1 | | | | | 2 | | | | | | | 3 | | | | 4 | | | | 5 | | | | 6 | | | | | | 7 | | |  | | | | | |
|  | |  | | |  | |  | | | | | | | | | | | | | | | | | | | | | | | | | | | | | | | | |  | | | | | | | | | |  |
|  | |  | | |  | |  | | | | | | | | | | | | | | | | | | | | | | | | | | | | | | | | |  | | | | | | | | | |  |
| 10 | | I intend to find out what the patient already knows or suspects | | | | | | |  | | | | 1 | | | | | 2 | | | | | | | 3 | | | | 4 | | | | 5 | | | | 6 | | | | | | 7 | | |  | | | | |
|  | |  | | |  | |  | | | | | | | | | | | | | | | | | | | | | | | | | | | | | | | | |  | | | | | | | | | |  |
|  | |  | | |  | |  | | | | | | | | | | | | | | | | | | | | | | | | | | | | | | | | |  | | | | | | | | | |  |
| 11 | In my practice I expect to find out what the patient already knows or suspects | | | | | | | |  | | | 1 | | | | 2 | | | | | | | 3 | | | | | 4 | | | | 5 | | | | 6 | | | | | | 7 | | |  | | | | | |
|  | |  | | |  | |  | | | | | | | | | | | | | | | | | | | | | | | | | | | | | | | | |  | | | | | | | | | |  |
|  | |  | | |  | |  | | | | | | | | | | | | | | | | | | | | | | | | | | | | | | | | |  | | | | | | | | | |  |
| 12 | | | I can rely on my colleagues in my Mental Health Team to find out what the patient already knows or suspects | | | | | | |  | | | | 1 | | | | | | 2 | | | | | | 3 | | | | 4 | | | | 5 | | | | 6 | | | | | | 7 | | |  | | |  |
|  | |  | |  | | | | |  | |  | | | |  | | | | | | |  | | | | |  | | | |  | | | |  | | | | | |  | | | | | | |  | |  |
|  | |  | |  | | | | |  | |  | | | |  | | | | | | |  | | | | |  | | | |  | | | |  | | | | | |  | | | | | | |  | |  |
| 13 | | | I feel that I have the skills I need to find out what the patient already knows or suspects | | | | | | |  | | | | 1 | | | | | 2 | | | | | | | 3 | | | | 4 | | | | 5 | | | | 6 | | | | | | 7 | | |  | | |  |
|  | |  | |  | | | | |  | |  | | | |  | | | | | | |  | | | | |  | | | |  | | | |  | | | | | |  | | | | | | |  | |  |
|  | |  | |  | | | | |  | |  | | | |  | | | | | | |  | | | | |  | | | |  | | | |  | | | | | |  | | | | | | |  | |  |
| 14 | | | It is easy for me to find out what the patient already knows or suspects | | | | | | |  | | | | 1 | | | | | 2 | | | | | | | 3 | | | | 4 | | | | 5 | | | | 6 | | | | | | 7 | | |  | | |  |
|  | |  | |  | | | | |  | |  | | | |  | | | | | | |  | | | | |  | | | |  | | | |  | | | | | |  | | | | | | |  | |  |
|  | |  | |  | | | | |  | |  | | | |  | | | | | | |  | | | | |  | | | |  | | | |  | | | | | |  | | | | | | |  | |  |
| 15 | | | I would feel uncomfortable whilst finding out what the patient already knows or suspects | | | | | | |  | | | | 1 | | | | | 2 | | | | | | | 3 | | | | 4 | | | | 5 | | | | 6 | | | | | | 7 | | |  | | |  |
|  | |  | | | |  | | |  | |  | | | |  | | | | | | |  | | | | |  | | | |  | | | |  | | | | | |  | | | | | | |  | |  |
|  | |  | | | |  | | |  | |  | | | |  | | | | | | |  | | | | |  | | | |  | | | |  | | | | | |  | | | | | | |  | |  |
| 16 | | | I would feel out of my depth whilst finding out what the patient already knows or suspects | | | | | | |  | | | | 1 | | | | | 2 | | | | | | | 3 | | | | 4 | | | | 5 | | | | 6 | | | | | | 7 | | |  | | |  |
|  | |  | | | |  | | |  | |  | | | |  | | | | | | |  | | | | |  | | | |  | | | |  | | | | | |  | | | | | | |  | |  |
|  | |  | | | |  | | |  | |  | | | |  | | | | | | |  | | | | |  | | | |  | | | |  | | | | | |  | | | | | | |  | |  |
| 17 | | | I would find it upsetting to find out what the patient already knows or suspects | | | | | | |  | | | | 1 | | | | | 2 | | | | | | | 3 | | | | 4 | | | | 5 | | | | 6 | | | | | | 7 | | |  | | |  |
|  | |  | | | |  | | |  | |  | | | |  | | | | | | |  | | | | |  | | | |  | | | |  | | | | | |  | | | | | | |  | |  |
|  | |  | | | |  | | |  | |  | | | |  | | | | | | |  | | | | |  | | | |  | | | |  | | | | | |  | | | | | | |  | |  |
|  | |  | | | |  | | |  | |  | | | |  | | | | | | |  | | | | |  | | | |  | | | |  | | | | | |  | | | | | | |  | |  |
|  | |  | | | |  | | |  | |  | | | |  | | | | | | |  | | | | |  | | | |  | | | |  | | | | | |  | | | | | | |  | |  |
|  | |  | | | |  | | |  | |  | | | |  | | | | | | |  | | | | |  | | | |  | | | |  | | | | | |  | | | | | | |  | |  |
|  | |  | | | |  | | |  | |  | | | |  | | | | | | |  | | | | |  | | | |  | | | |  | | | | | |  | | | | | | |  | |  |
|  | |  | | | |  | | |  | |  | | | |  | | | | | | |  | | | | |  | | | |  | | | |  | | | | | |  | | | | | | |  | |  |
|  | |  | | | |  | | |  | |  | | | |  | | | | | | |  | | | | |  | | | |  | | | |  | | | | | |  | | | | | | |  | |  |

| **In relation to finding out what the patient already knows or suspects about their diagnosis** |
| --- |

| **I am confident that I can find out what the patient already knows or suspects…** | | | | | | | | | | |  | | |  | | | | |  | | | |  | | |  | | |  | | | | | |  | | | | |  | |  | |
| --- | --- | --- | --- | --- | --- | --- | --- | --- | --- | --- | --- | --- | --- | --- | --- | --- | --- | --- | --- | --- | --- | --- | --- | --- | --- | --- | --- | --- | --- | --- | --- | --- | --- | --- | --- | --- | --- | --- | --- | --- | --- | --- | --- |
|  | | |  | | | |  | |  | **Strongly Disagree** | | | | | | | |  | | | | | | |  | | |  | | | | | **Strongly**  **Agree** | | | | | | | |  | | |
|  | | |  | | | |  | |  | | | | | | | | | | | | | | | | | | | | | | | |  | | | | | | | |  | | |
| 18 | | ….when I have sufficient time with the patient | | | | | | | |  | | 1 | | | | 2 | | | | | 3 | | | 4 | | | 5 | | | | 6 | | | | | 7 | |  | | | | | |
|  | |  | | | |  | |  | | | | | | | | | | | | | | | | | | | | | | | | | |  | | | | | | | |  | |
|  | |  | | | |  | |  | | | | | | | | | | | | | | | | | | | | | | | | | |  | | | | | | | |  | |
| 19 | | ….when a carer or relative is present | | | | | | | |  | | | 1 | | | | 2 | | | | | 3 | | | 4 | | | 5 | | | | 6 | | | | | 7 | |  | | | | |
|  | |  | | | |  | |  | | | | | | | | | | | | | | | | | | | | | | | | | |  | | | | | | | |  | |
|  | |  | | | |  | |  | | | | | | | | | | | | | | | | | | | | | | | | | |  | | | | | | | |  | |
| 20 | ….when the patient is mildly impaired | | | | | | | | |  | | 1 | | | 2 | | | | | 3 | | | | 4 | | | 5 | | | | 6 | | | | | 7 | |  | | | | | |
|  | |  | | | |  | |  | | | | | | | | | | | | | | | | | | | | | | | | | |  | | | | | | | |  | |
|  | |  | | | |  | |  | | | | | | | | | | | | | | | | | | | | | | | | | |  | | | | | | | |  | |
| 21 | ….when the patient is severely impaired | | | | | | | | |  | | 1 | | | 2 | | | | | 3 | | | | 4 | | | 5 | | | | 6 | | | | | 7 | |  | | | | | |
|  | | |  |  |  | | | | | | | | | | | | | | | | | | | | | | | | |  | | | | | | | | | | | | |  |
|  | | |  |  |  | | | | | | | | | | | | | | | | | | | | | | | | |  | | | | | | | | | | | | |  |

|  |  |  |  |  | |  | |  |  | |
| --- | --- | --- | --- | --- | --- | --- | --- | --- | --- | --- |
|  |  |  |  |  | |  | |  |  | |
|  | **Thinking about my role in the process of disclosing a diagnosis of dementia:** | | |  |  | |  | | |  |

| 22 | | What patients with dementia think I should do is very important to me | | |  | 1 | | | 2 | | | 3 | | 4 | | 5 | | 6 | | | 7 | |  | | |
| --- | --- | --- | --- | --- | --- | --- | --- | --- | --- | --- | --- | --- | --- | --- | --- | --- | --- | --- | --- | --- | --- | --- | --- | --- | --- |
|  | |  |  |  | | | | | | | | | | | | | | | |  | | | | |  |
|  | |  |  |  | | | | | | | | | | | | | | | |  | | | | |  |
| 23 | | What carers and relatives of patients with dementia think I should do is very important to me | | |  | | 1 | | | 2 | | | 3 | | 4 | | 5 | | 6 | | | 7 | |  | |
|  | |  |  |  | | | | | | | | | | | | | | | |  | | | | |  |
|  | |  |  |  | | | | | | | | | | | | | | | |  | | | | |  |
| 24 | What members of my Mental Health Team think I should do is very important to me | | | |  | 1 | | 2 | | | 3 | | | 4 | | 5 | | 6 | | | 7 | |  | | |
|  | |  |  |  | | | | | | | | | | | | | | | |  | | | | |  |
|  | |  |  |  | | | | | | | | | | | | | | | |  | | | | |  |

|  |  | |  |  |  |  |  |  |  |  |  |  |
| --- | --- | --- | --- | --- | --- | --- | --- | --- | --- | --- | --- | --- |
|  |  | |  |  |  |  |  |  |  |  |  |  |
|  |  | |  |  |  |  |  |  |  |  |  |  |
| ***SECTION B* USING THE ACTUAL WORDS ‘DEMENTIA’ OR ‘ALZHEIMER’S DISEASE’ WHEN TALKING TO THE PATIENT**  **These questions are about the words you use**  **when telling patients that they have dementia or soon afterwards** | | | | | | | | | | | | |
|  | |  | | | | | | | | | | |
|  | | **I think that, overall, using the actual words ‘dementia’ or ‘Alzheimer’s disease’ when talking to the patient…** | | | | | | | | | | |
|  | |  | | | | | | | | | | |

| 1 | …is harmful to the patient | | |  | 1 | | 2 | | 3 | | 4 | | 5 | | 6 | | 7 | | beneficial to the patient | | | |  |
| --- | --- | --- | --- | --- | --- | --- | --- | --- | --- | --- | --- | --- | --- | --- | --- | --- | --- | --- | --- | --- | --- | --- | --- |
|  |  |  |  |  |  |  | |  | |  | |  | |  | |  | |  | | | |  | |
|  |  |  |  |  |  |  | |  | |  | |  | |  | |  | |  | | | |  | |
| 2 | …is the wrong thing to do | | |  | 1 | | 2 | | 3 | | 4 | | 5 | | 6 | | 7 | | the right thing to do | | | |  |
|  |  |  |  |  |  |  | |  | |  | |  | |  | |  | |  | | | |  | |
|  |  |  |  |  |  |  | |  | |  | |  | |  | |  | |  | | | |  | |
| 3 | …is not distressing to the  patient | | |  | 1 | | 2 | | 3 | | 4 | | 5 | | 6 | | 7 | | distressing to the patient | | | |  |
|  |  |  |  |  |  |  | |  | |  | |  | |  | |  | |  | | | |  | |
|  |  |  |  |  |  |  | |  | |  | |  | |  | |  | |  | | | |  | |
| 4 | …is a poor use of my time | | |  | 1 | | 2 | | 3 | | 4 | | 5 | | 6 | | 7 | | a good use of my time | | | |  |
|  |  | |  |  |  |  | |  | |  | |  | |  | |  | |  | | | |  | |
|  |  | |  |  |  |  | |  | |  | |  | |  | |  | |  | | | |  | |
| 5 | …results in the patient being  stigmatised | | |  | 1 | | 2 | | 3 | | 4 | | 5 | | 6 | | 7 | | does not result in the patient being stigmatised | | | |  |
|  |  |  |  |  |  |  | |  | |  | |  | |  | |  | |  | | | |  | |
|  |  |  |  |  |  |  | |  | |  | |  | |  | |  | |  | | | |  | |
| 6 | …makes it easier for the patient  to understand what is  happening to them | | |  | 1 | | 2 | | 3 | | 4 | | 5 | | 6 | | 7 | | makes it more difficult for the patient to understand what is happening to them | | | |  |
|  |  |  |  |  |  |  | |  | |  | |  | |  | |  | |  | | |  | | |
|  |  |  |  |  |  |  | |  | |  | |  | |  | |  | |  | | |  | | |
| 7 | …discourages the patient from  planning for the future | | |  | 1 | | 2 | | 3 | | 4 | | 5 | | 6 | | 7 | | | encourages the patient to plan for the future | | |  |
|  |  |  |  |  |  |  | |  | |  | |  | |  | |  | |  | | | |  | |
|  |  |  |  |  |  |  | |  | |  | |  | |  | |  | |  | | | |  | |

|  |  |  |  |  |  |  |  |  |  |  |  |  |
| --- | --- | --- | --- | --- | --- | --- | --- | --- | --- | --- | --- | --- |

| **In relation to using the actual words ‘dementia’ or ‘Alzheimer’s disease’ when talking to the patient** |
| --- |

|  |  |  | **Strongly Disagree** | |  | |  | |  | | **Strongly Agree** | | |
| --- | --- | --- | --- | --- | --- | --- | --- | --- | --- | --- | --- | --- | --- |
| 8 | Generally, patients with dementia think that I should use the actual words ‘dementia’ or ‘Alzheimer’s disease’ when talking to them |  | 1 | 2 | | 3 | | 4 | | 5 | | 6 | 7 |
|  |  | | | | | | | | | | | | |
|  |  | | | | | | | | | | | | |
| 9 | Generally, carers and relatives of patients with dementia think that I should use the actual words ‘dementia’ or ‘Alzheimer’s disease’ when talking to the patient |  | 1 | 2 | | 3 | | 4 | | 5 | | 6 | 7 |
|  |  | | | | | | | | | | | | |
|  |  | | | | | | | | | | | | |
| 10 | Members of my Mental Health Team would approve of my using the actual words ‘dementia’ or ‘Alzheimer’s disease’ when talking to the patient |  | 1 | 2 | | 3 | | 4 | | 5 | | 6 | 7 |
|  |  | | | | | | | | | | | | |
| 11 | Other people who are important to me professionally would approve of my using the actual words ‘dementia’ or ‘Alzheimer’s disease’ when talking to the patient |  | 1 | 2 | | 3 | | 4 | | 5 | | 6 | 7 |
|  |  | | | | | | | | | | | | |
|  |  | | | | | | | | | | | | |
| 12 | The decision whether or not to use the actual words ‘dementia’ or ‘Alzheimer’s disease’ when talking to the patient is beyond my control |  | 1 | 2 | | 3 | | 4 | | 5 | | 6 | 7 |
|  |  | | | | | | | | | | | | |
|  |  | | | | | | | | | | | | |
| 13 | I intend to use the actual words ‘dementia’ or ‘Alzheimer’s disease’ when talking to the patient |  | 1 | 2 | | 3 | | 4 | | 5 | | 6 | 7 |
|  |  | | | | | | | | | | | | |
|  |  | | | | | | | | | | | | |
| 14 | In my practice I expect to use the actual words ‘dementia’ or ‘Alzheimer’s disease’ when talking to the patient |  | 1 | 2 | | 3 | | 4 | | 5 | | 6 | 7 |
|  |  | | | | | | | | | | | | |
| 15 | I can rely on my colleagues in my Mental Health Team to use the actual words ‘dementia’ or ‘Alzheimer’s disease’ when talking to the patient |  | 1 | 2 | | 3 | | 4 | | 5 | | 6 | 7 |
|  |  | | | | | | | | | | | | |
|  |  | | | | | | | | | | | | |
| 16 | It is easy for me to use the actual words ‘dementia’ or ‘Alzheimer’s disease’ when talking to the patient |  | 1 | 2 | | 3 | | 4 | | 5 | | 6 | 7 |
|  |  | | | | | | | | | | | | |
|  |  | | | | | | | | | | | | |
| 17 | I would feel uncomfortable whilst using the words ‘dementia’ or ‘Alzheimer’s disease’ when talking to the patient |  | 1 | 2 | | 3 | | 4 | | 5 | | 6 | 7 |
|  |  | | | | | | | | | | | | |
|  |  | | | | | | | | | | | | |
| 18 | I would feel out of my depth whilst using the words ‘dementia’ or ‘Alzheimer’s disease’ when talking to the patient |  | 1 | 2 | | 3 | | 4 | | 5 | | 6 | 7 |
|  |  | | | | | | | | | | | | |
|  |  | | | | | | | | | | | | |
| 19 | I would find it upsetting to use the words ‘dementia’ or ‘Alzheimer’s disease’ when talking to the patient |  | 1 | 2 | | 3 | | 4 | | 5 | | 6 | 7 |

| **In relation to using the actual words ‘dementia’ or ‘Alzheimer’s disease’ when talking to the patient** | | | | | | | | | | | | |  |
| --- | --- | --- | --- | --- | --- | --- | --- | --- | --- | --- | --- | --- | --- |
|  |  |  |  | |  |  |  |  |  |  |  |  | |
|  |  |  |  | |  |  |  |  |  |  |  |  | |
| **I am confident that I can use the words ‘dementia’ or ‘Alzheimer’s disease’ when talking to the patient** **…** | | | |  | | | | | | | |  | |

|  |  | |  | | **Strongly Disagree** | |  | |  | |  | | **Strongly Agree** | | | | |  |
| --- | --- | --- | --- | --- | --- | --- | --- | --- | --- | --- | --- | --- | --- | --- | --- | --- | --- | --- |
|  |  | | |  | | | | | | | | | | |  | | | |
| 20 | …when the patient thinks that their problems  are just due to old age |  |  | | 1 | 2 | | 3 | | 4 | | 5 | | 6 | | 7 | | |
|  |  |  |  | |  |  | |  | |  | |  | |  | |  | | |
|  |  |  |  | |  |  | |  | |  | |  | |  | |  | | |
| 21 | …when I have sufficient time with the patient |  |  | | 1 | 2 | | 3 | | 4 | | 5 | | 6 | | 7 | | |
|  |  |  |  | |  |  | |  | |  | |  | |  | |  | | |
|  |  |  |  | |  |  | |  | |  | |  | |  | |  | | |
| 23 | …when a carer or relative is present |  |  | | 1 | 2 | | 3 | | 4 | | 5 | | 6 | | 7 | | |
|  |  |  |  | |  |  | |  | |  | |  | |  | |  | | |
|  |  |  |  | |  |  | |  | |  | |  | |  | |  | | |
| 24 | …when the patient does not want to know the  diagnosis |  |  | | 1 | 2 | | 3 | | 4 | | 5 | | 6 | | 7 | | |
|  |  |  |  | |  |  | |  | |  | |  | |  | |  | | |
|  |  |  |  | |  |  | |  | |  | |  | |  | |  | | |
| 25 | …when the patient is mildly impaired |  |  | | 1 | 2 | | 3 | | 4 | | 5 | | 6 | | 7 | | |
|  |  |  |  | |  |  | |  | |  | |  | |  | |  | | |
|  |  |  |  | |  |  | |  | |  | |  | |  | |  | | |
| 26 | ...when the patient is severely impaired |  |  | | 1 | 2 | | 3 | | 4 | | 5 | | 6 | | 7 | | |
|  |  |  |  | |  |  | |  | |  | |  | |  | |  | | |
|  |  |  |  | |  |  | |  | |  | |  | |  | |  | | |
| 27 | …when the carer has requested that the  diagnosis be withheld |  |  | | 1 | 2 | | 3 | | 4 | | 5 | | 6 | | 7 | | |
|  |  |  |  | |  |  | |  | |  | |  | |  | |  | | |
|  |  |  |  | |  |  | |  | |  | |  | |  | |  | | |
| 28 | …when appropriate support for the patient is  available shortly afterwards |  |  | | 1 | 2 | | 3 | | 4 | | 5 | | 6 | | 7 | | |
|  |  |  |  | |  |  | |  | |  | |  | |  | |  |  | |
|  |  |  |  | |  |  | |  | |  | |  | |  | |  |  | |
|  |  |  |  | |  |  | |  | |  | |  | |  | |  |  | |
|  |  |  |  | |  |  | |  | |  | |  | |  | |  |  | |
|  |  |  |  | |  |  | |  | |  | |  | |  | |  |  | |
|  |  |  |  | |  |  | |  | |  | |  | |  | |  |  | |
|  |  |  |  | |  |  | |  | |  | |  | |  | |  |  | |
|  |  |  |  | |  |  | |  | |  | |  | |  | |  |  | |
|  |  |  |  | |  |  | |  | |  | |  | |  | |  |  | |
|  |  |  |  | |  |  | |  | |  | |  | |  | |  |  | |
|  |  |  |  | |  |  | |  | |  | |  | |  | |  |  | |
|  |  |  |  | |  |  | |  | |  | |  | |  | |  |  | |
|  |  |  |  | |  |  | |  | |  | |  | |  | |  |  | |
|  |  |  |  | |  |  | |  | |  | |  | |  | |  |  | |

| ***SECTION C* EXPLORING WHAT THE DIAGNOSIS MEANS TO THE PATIENT**  **(ie their BELIEFS & RELATED EMOTIONS)**  **These questions are about exploring the patient’s response**  **AFTER they have been told the diagnosis** | |
| --- | --- |
|  |  |
|  | **I think that, overall, exploring what the diagnosis means to the patient is …** |

|  |  |
| --- | --- |

| 1 | …harmful to the patient |  | 1 | 2 | 3 | 4 | 5 | 6 | 7 |  | beneficial to the patient |
| --- | --- | --- | --- | --- | --- | --- | --- | --- | --- | --- | --- |
|  |  |  |  |  |  |  |  |  |  |  |  |
|  |  |  |  |  |  |  |  |  |  |  |  |
| 2 | …the wrong thing to do |  | 1 | 2 | 3 | 4 | 5 | 6 | 7 |  | the right thing to do |
|  |  |  |  |  |  |  |  |  |  |  |  |
|  |  |  |  |  |  |  |  |  |  |  |  |
| 3 | …not distressing for the  patient |  | 1 | 2 | 3 | 4 | 5 | 6 | 7 |  | distressing for the patient |
|  |  |  |  |  |  |  |  |  |  |  |  |
|  |  |  |  |  |  |  |  |  |  |  |  |
| 4 | …a poor use of my time |  | 1 | 2 | 3 | 4 | 5 | 6 | 7 |  | good use of my time |
|  |  |  |  |  |  |  |  |  |  |  |  |
|  |  |  |  |  |  |  |  |  |  |  |  |
| 5 | …results in the patient  being stigmatised |  | 1 | 2 | 3 | 4 | 5 | 6 | 7 |  | does not result in the patient  being stigmatised |
|  |  |  |  |  |  |  |  |  |  |  |  |
|  |  |  |  |  |  |  |  |  |  |  |  |
| 6 | …makes it easier for the  patient to understand what is happening to them |  | 1 | 2 | 3 | 4 | 5 | 6 | 7 |  | makes it more difficult for the patient to understand what is happening to them |
|  |  |  |  |  |  |  |  |  |  |  |  |
|  |  |  |  |  |  |  |  |  |  |  |  |
| 7 | …discourages the patient  from planning for the future |  | 1 | 2 | 3 | 4 | 5 | 6 | 7 |  | encourages the patient to plan  for the future |

| **In relation to exploring what the diagnosis means to the patient** |
| --- |

|  |  |  | **Strongly Disagree** |  |  |  | **Strongly Agree** |
| --- | --- | --- | --- | --- | --- | --- | --- |

| 8 | | | Generally, patients with dementia think that I should explore what the diagnosis means to them | | | | | |  | | 1 | | | | 2 | | | | 3 | | | 4 | | | 5 | | | 6 | | | | | 7 | |  | | | | |
| --- | --- | --- | --- | --- | --- | --- | --- | --- | --- | --- | --- | --- | --- | --- | --- | --- | --- | --- | --- | --- | --- | --- | --- | --- | --- | --- | --- | --- | --- | --- | --- | --- | --- | --- | --- | --- | --- | --- | --- |
|  | | |  |  | | | |  | | | | | | | | | | | | | | | | | | | | | | |  | | | | | | | |  |
|  | | |  |  | | | |  | | | | | | | | | | | | | | | | | | | | | | |  | | | | | | | |  |
| 9 | | | Generally, carers and relatives of patients with dementia think that I should explore what the diagnosis means to the patient | | | | | |  | | | 1 | | | | 2 | | | | 3 | | | 4 | | | 5 | | | 6 | | | | | 7 | |  | | | |
|  | | |  |  | | | |  | | | | | | | | | | | | | | | | | | | | | | |  | | | | | | | |  |
|  | | |  |  | | | |  | | | | | | | | | | | | | | | | | | | | | | |  | | | | | | | |  |
| 10 | | | Members of my Mental Health Team would approve of my exploring what the diagnosis means to the patient | | | | | |  | | 1 | | | 2 | | | | 3 | | | | 4 | | | 5 | | | 6 | | | | | 7 | |  | | | | |
|  | | |  |  | | | |  | | | | | | | | | | | | | | | | | | | | | | |  | | | | | | | |  |
|  | | |  |  | | | |  | | | | | | | | | | | | | | | | | | | | | | |  | | | | | | | |  |
| 11 | | | Other people who are important to me professionally would approve of my exploring what the diagnosis means to the patient | | | | | |  | | 1 | | | 2 | | | | 3 | | | | 4 | | | 5 | | | 6 | | | | | 7 | |  | | | | |
|  | |  | | | |  |  | | |  | | |  | | | |  | | | |  | | |  | | |  | | |  | |  | | | | | |  | |
|  | |  | | | |  |  | | |  | | |  | | | |  | | | |  | | |  | | |  | | |  | |  | | | | | |  | |
| 12 | | | The decision whether or not to explore what the diagnosis means to the patient is beyond my control | | | | | |  | | 1 | | | | 2 | | | | 3 | | | 4 | | | 5 | | | 6 | | | | | 7 | |  | | | | |
|  | | |  |  | | | |  | | | | | | | | | | | | | | | | | | | | | | |  | | | | | | | |  |
|  | | |  |  | | | |  | | | | | | | | | | | | | | | | | | | | | | |  | | | | | | | |  |
| 13 | | | I intend to explore what the diagnosis means to the patient | | | | | |  | | | 1 | | | | 2 | | | | 3 | | | 4 | | | 5 | | | 6 | | | | | 7 | |  | | | |
|  | | |  |  | | | |  | | | | | | | | | | | | | | | | | | | | | | |  | | | | | | | |  |
|  | | |  |  | | | |  | | | | | | | | | | | | | | | | | | | | | | |  | | | | | | | |  |
| 14 | | | In my practice I expect to explore what the diagnosis means to the patient | | | | | |  | | 1 | | | 2 | | | | 3 | | | | 4 | | | 5 | | | 6 | | | | | 7 | |  | | | | |
|  | | |  |  | | | |  | | | | | | | | | | | | | | | | | | | | | | |  | | | | | | | |  |
|  | | |  |  | | | |  | | | | | | | | | | | | | | | | | | | | | | |  | | | | | | | |  |
| 15 | | | I can rely on my colleagues in my Mental Health Team to explore what the diagnosis means to the patient | | | | | |  | | 1 | | | 2 | | | | 3 | | | | 4 | | | 5 | | | 6 | | | | | 7 | |  | | | | |
|  |  | | | |  | | | |  | | | | | | | | | | | | | | | | | | | | | | | | | | | |  | | |
|  |  | | | |  | | | |  | | | | | | | | | | | | | | | | | | | | | | | | | | | |  | | |
| 16 | | | In feel that I have the skills I need to explore what the diagnosis means to the patient | | | | | |  | | 1 | | | | 2 | | | | 3 | | | 4 | | | 5 | | | 6 | | | | | 7 | |  | | | | |
|  | | |  |  | | | |  | | | | | | | | | | | | | | | | | | | | | | |  | | | | | | | |  |
|  | | |  |  | | | |  | | | | | | | | | | | | | | | | | | | | | | |  | | | | | | | |  |
| 17 | | | It is easy for me to explore what the diagnosis means to the patient | | | | | |  | | | 1 | | | | 2 | | | | 3 | | | 4 | | | 5 | | | 6 | | | | | 7 | |  | | | |
|  | | |  |  | | | |  | | | | | | | | | | | | | | | | | | | | | | |  | | | | | | | |  |
|  | | |  |  | | | |  | | | | | | | | | | | | | | | | | | | | | | |  | | | | | | | |  |
| 18 | | | I would feel uncomfortable whilst exploring what the diagnosis means to the patient | | | | | |  | | 1 | | | 2 | | | | 3 | | | | 4 | | | 5 | | | 6 | | | | | 7 | |  | | | | |
|  | | |  |  | | | |  | | | | | | | | | | | | | | | | | | | | | | |  | | | | | | | |  |
|  | | |  |  | | | |  | | | | | | | | | | | | | | | | | | | | | | |  | | | | | | | |  |
| 19 | | | I would feel out of my depth whilst exploring what the diagnosis means to the patient | | | | | |  | | 1 | | | 2 | | | | 3 | | | | 4 | | | 5 | | | 6 | | | | | 7 | |  | | | | |
|  | | |  | | | | | |  | |  | | |  | | | |  | | | |  | | |  | | |  | | | | |  | |  | | | | |
|  | | |  | | | | | |  | |  | | |  | | | |  | | | |  | | |  | | |  | | | | |  | |  | | | | |
| 20 | | | I would find it upsetting to explore what the diagnosis meant to the patient | | | | | |  | | 1 | | | 2 | | | | 3 | | | | 4 | | | 5 | | | 6 | | | | | 7 | |  | | | | |
|  | |  | | | |  |  | | |  | | |  | | | |  | | | |  | | |  | | |  | | |  | |  | | | | | |  | |
|  | |  | | | |  |  | | |  | | |  | | | |  | | | |  | | |  | | |  | | |  | |  | | | | | |  | |

| **In relation to exploring what the diagnosis means to the patient** |
| --- |

| **I am confident that I can explore what the diagnosis means to the patient …** |  |  |  |  |  |  |  |  |
| --- | --- | --- | --- | --- | --- | --- | --- | --- |
|  |  |  |  |  |  |  |  |  |

|  |  | |  | | **Strongly Disagree** | |  | |  | |  | | **Strongly Agree** | | | |  |
| --- | --- | --- | --- | --- | --- | --- | --- | --- | --- | --- | --- | --- | --- | --- | --- | --- | --- |
|  |  | | |  | | | | | | | | | | |  | | |
| 21 | … when I have sufficient time with the patient |  |  | | 1 | 2 | | 3 | | 4 | | 5 | | 6 | | 7 | |
|  |  |  |  | |  |  | |  | |  | |  | |  | |  | |
|  |  |  |  | |  |  | |  | |  | |  | |  | |  | |
| 22 | … when a carer or relative is present |  |  | | 1 | 2 | | 3 | | 4 | | 5 | | 6 | | 7 | |
|  |  |  |  | |  |  | |  | |  | |  | |  | |  | |
|  |  |  |  | |  |  | |  | |  | |  | |  | |  | |
| 23 | … when the patient is mildly impaired |  |  | | 1 | 2 | | 3 | | 4 | | 5 | | 6 | | 7 | |
|  |  |  |  | |  |  | |  | |  | |  | |  | |  | |
|  |  |  |  | |  |  | |  | |  | |  | |  | |  | |
| 24 | … when the patient is severely impaired |  |  | | 1 | 2 | | 3 | | 4 | | 5 | | 6 | | 7 | |
|  |  |  |  | |  |  | |  | |  | |  | |  | |  | |
|  |  |  |  | |  |  | |  | |  | |  | |  | |  | |
| 25 | … when appropriate support for the patient is available shortly afterwards |  |  | | 1 | 2 | | 3 | | 4 | | 5 | | 6 | | 7 | |
|  |  |  |  | |  |  | |  | |  | |  | |  | |  | |
|  |  |  |  | |  |  | |  | |  | |  | |  | |  | |

| ***SECTION D* REFLECTING ON CURRENT PRACTICE**    **The questions in this section are concerned with how far you and other team members are able to carry out these behaviours in practice** |
| --- |

| We would like you to focus on the last five patients you saw with a new diagnosis of dementia. Please think about the extent to which you were able to carry out each behaviour with each patient; then write the actual number of patients in the appropriate columns. For example, if you did not find out what two patients suspected, but found out to some extent with one patient and explored the suspicions of the remaining two patients in detail, you would write the numbers 2, 1 and 2 on the dotted lines in the first column. |
| --- |

1 Of the last five patients you saw with a new diagnosis of dementia, how many of these did you personally:

|  | Find out what the  patient already knew or  suspected about the  diagnosis | Use the actual words  ‘dementia’ or ‘Alzheimer’s  disease’ when talking  to the patient | Explore what the  diagnosis means  to the patient |
| --- | --- | --- | --- |

| Not at all | ………. | ………. | ………. |
| --- | --- | --- | --- |

| To some extent | ………. | ………. | ………. |
| --- | --- | --- | --- |

| Fully | ………. | ………. | ………. |
| --- | --- | --- | --- |

|  | *Total=5* | *Total=5* | *Total=5* |
| --- | --- | --- | --- |
|  |  |  |  |

*Any comments:*

2 Of the last five patients you saw with a new diagnosis of dementia, how many of these did any other team members:

|  | Find out what the  patient already knew or  suspected about the  diagnosis | Use the actual words  ‘dementia’ or ‘Alzheimer’s  disease’ when talking  to the patient | Explore what the  diagnosis means  to the patient |
| --- | --- | --- | --- |

| Not at all | ………. | ………. | ………. |
| --- | --- | --- | --- |

| To some extent | ………. | ………. | ………. |
| --- | --- | --- | --- |

| Fully | ………. | ………. | ………. |
| --- | --- | --- | --- |

| Not sure | ………. | ………. | ………. |
| --- | --- | --- | --- |

|  | *Total=5* | *Total=5* | *Total=5* |
| --- | --- | --- | --- |
|  |  |  |  |

*Any comments:*

| ***SECTION E*  What professionals know about longer term outcomes of dementia may influence what they say to patients and their carers. This section contains factual questions relating to prognosis.**  **Some of these questions might be difficult to answer but**  **we are interested in what you think** |
| --- |

1 What percentage of people with Alzheimer’s disease is admitted to permanent long term care?

0-33% 1

34-66% 2

67-100% 3

Not sure or good evidence lacking 4

2 Although the rate of progression of Alzheimer's disease is variable, the average life expectancy after onset is:

0 to 5 years 1

6 to 10 years 2

11 to 15 years 3

16 + years 4

Not sure or good evidence lacking 5

3 Although the rate of progression of vascular dementia is variable, the average life expectancy after onset is:

0 to 5 years 1

6 to 10 years 2

11 to 15 years 3

16 + years 4

Not sure or good evidence lacking 5

4 What percentage of people with dementia die of (rather than with) their dementia?

0-33% 1

34-66% 2

67-100% 3

Not sure or good evidence lacking 4

5 Do you feel you need additional training on the process of disclosing the diagnosis to patients with dementia?

Yes 1

No 2

**IF YES,** please describe your training needs

| 6 | In your Mental Health Team/Memory Clinic, who does the following?  *(Please circle* ***all*** *that apply)* | | | | |
| --- | --- | --- | --- | --- | --- |
|  |  |  | Finds out what the patient already knows or suspects | Uses the actual words ‘dementia’ or ‘Alzheimer’s disease’ when talking to the patient | Explores what the diagnosis means to the patient |
|  |  |  |  |  |  |
| A | Psychiatrist |  | 1 | 2 | 3 |
|  |  |  |  |  |  |
| B | Social worker |  | 1 | 2 | 3 |
|  |  |  |  |  |  |
| C | Clinical psychologist |  | 1 | 2 | 3 |
|  |  |  |  |  |  |
| D | Community psychiatric nurse |  | 1 | 2 | 3 |
|  |  |  |  |  |  |
| E | Occupational therapist |  | 1 | 2 | 3 |
|  |  |  |  |  |  |
| F | Care or nursing assistant |  | 1 | 2 | 3 |
|  |  |  |  |  |  |
| G | In-patient or day hospital nurse |  | 1 | 2 | 3 |
|  |  |  |  |  |  |
| H | Other (please give details)  ………………………………………  …………………………………… |  | 1 | 2 | 3 |

7 Please write any additional comments below

**Thank you for your time**

**Please place your completed questionnaire in the**

**reply paid envelope provided and return to**

**Jan Lecouturier, Centre for Health Services Research,**

**University of Newcastle, 21 Claremont Place,**

**Newcastle upon Tyne, NE2 4AA**
